# Splenic hamartoma in two related patients with *BAP1* tumour predisposition syndrome caused by a novel germline *BAP1* p.(Gly128Arg) missense variant

**DOI:** 10.1007/s10689-026-00538-3

**Published:** 2026-02-19

**Authors:** Kristjan Ari Ragnarsson, Gloria Garcia, Jon Gunnlaugur Jonasson, Gudny Anna Arnadottir, Sigrun Edda Reykdal, Reynir Arngrimsson, Sigurdis Haraldsdottir, Jon Johannes Jonsson

**Affiliations:** 1 Department of Genetics and Molecular Medicine, Landspitali University Hospital, Reykjavik, Iceland; 2 Department of Pathology, Landspitali University Hospital, Reykjavik, Iceland; 3 Faculty of Medicine, University of Iceland, Reykjavik, Iceland; 4 deCODE genetics/Amgen, Inc., Reykjavik, Iceland; 5 Department of Hematology, Landspitali University Hospital, Reykjavik, Iceland; 6 Department of Oncology, Landspitali University Hospital, Reykjavik, Iceland

**Keywords:** *BAP1* tumour predisposition syndrome, Splenic hamartoma, *BAP1* missense variant, BAP1 immunohistochemistry

## Abstract

*BAP1* tumour predisposition syndrome (*BAP1*-TPDS) is a hereditary cancer syndrome caused by heterozygous pathogenic germline variants in *BAP1*. *BAP1*-TPDS is associated with an increased risk for various malignant tumours, the core of which is uveal and cutaneous melanoma, malignant mesothelioma, and renal cell carcinoma. In *BAP1*-TPDS, the majority of disease-causing *BAP1* variants are null variants, although missense variants have been reported. We report a patient with *BAP1*-TPDS caused by the novel germline *BAP1* missense variant NM_004656.4:c.382G > A, p.(Gly128Arg). The patient developed *BAP1*-inactivated melanocytic tumours, clear-cell renal cell carcinoma, and splenic hamartoma. An incidental splenic hamartoma was detected in existing tissue slides from the patient’s deceased first-degree relative, who was an obligate carrier for *BAP1*-TPDS. In both splenic hamartomas, loss of BAP1 nuclear staining was detected in a subset of cells on immunohistochemistry. To our knowledge, this is the first report with immunohistochemical data supporting biallelic loss of *BAP1* as a contributing step in the development of a splenic hamartoma in a patient with *BAP1*-TPDS. It supports expanding the tumour spectrum of *BAP1*-TPDS to splenic hamartoma and possibly other benign splenic tumours.

**Supplementary Information:**

The online version contains supplementary material available at 10.1007/s10689-026-00538-3.

## Introduction

The BRCA1-associated protein 1 gene (*BAP1*; OMIM *603089) is a tumour suppressor gene that encodes a deubiquitinase involved in DNA repair and epigenetic regulation [1, 2]. Heterozygous loss-of-function pathogenic germline *BAP1* variants are associated with autosomal dominant *BAP1* tumour predisposition syndrome (*BAP1*-TPDS; OMIM #614327).

Core tumours of *BAP1*-TPDS include uveal and cutaneous melanoma, pleural and peritoneal mesothelioma, renal cell carcinoma (RCC; predominantly clear cell), and benign *BAP1*-inactivated melanocytic tumours (BIMTs). BIMTs rarely transform to melanoma. Other tumours include meningioma, basal cell carcinoma, and cholangiocarcinoma [3, 4]. In a case series, Miranda et al. (2024) provided the first indication of an association between *BAP1*-TPDS and benign splenic lesions, including a suspected splenic hamartoma [5].

The mechanism of tumour formation in *BAP1*-TPDS involves a somatic variant in the second *BAP1* allele or loss of heterozygosity, leading to biallelic *BAP1* loss. Immunohistochemistry (IHC) on *BAP1*-deficient tumours shows absent nuclear staining for BAP1, reflecting loss of nuclear BAP1 expression [3].

Lifetime risks are estimated to be 20–25% for uveal/cutaneous melanoma and mesothelioma, and lower for other tumours. Overall lifetime risk for at least one tumour is as high as 85%. The risk estimates may be inflated due to ascertainment bias [3, 6]. The increased risk for malignant pleural mesothelioma may partly be mediated by increased asbestos sensitivity [7].

We describe a patient with a previously unreported germline *BAP1* missense variant who developed BIMTs, clear cell RCC, and splenic hamartoma. On IHC, loss of nuclear BAP1 staining was observed in the BIMTs and RCC, and in a subset of cells within the splenic hamartoma. This report suggests that splenic hamartomas represent a benign manifestation of the tumour spectrum observed in *BAP1*-TPDS, consistent with prior reports.

## Subject, materials, and methods

### Case presentation

**Fig. 1:**
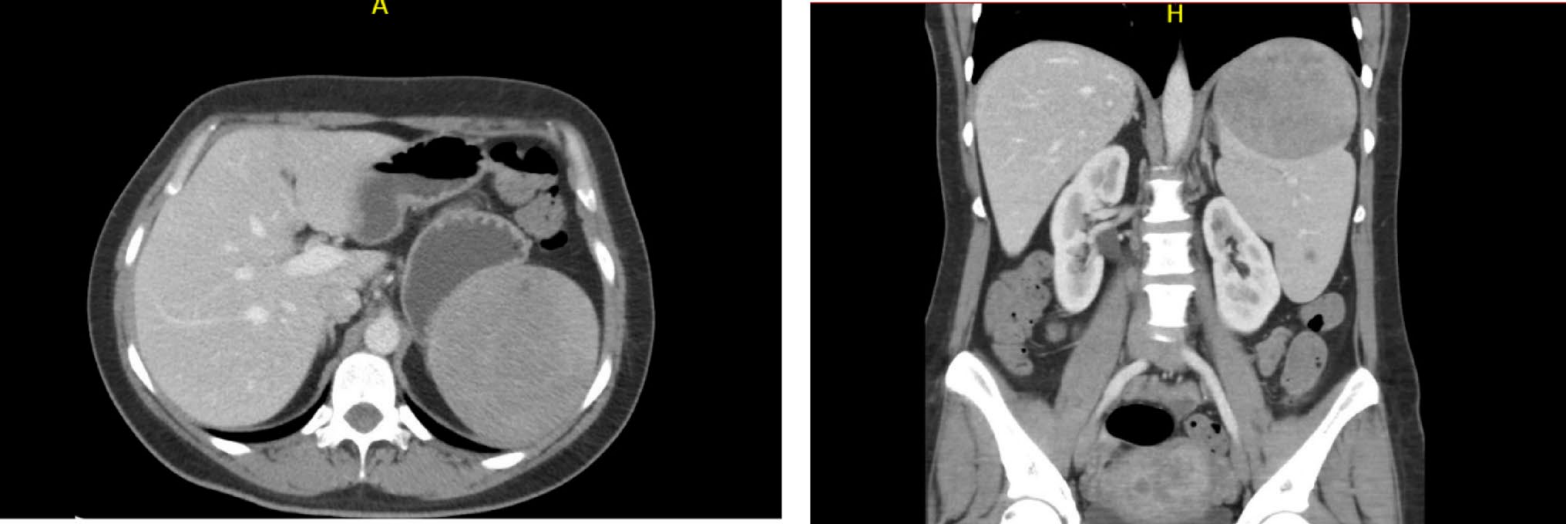
CT scan of the patient showing the splenic hamartoma above the upper pole of the left kidney in coronal (left) and axial (right) planes

The proband (hereafter ‘the patient’) is a female with a complex tumour history. She was diagnosed with uterine leiomyoma at age 34, followed by splenic hamartoma (Fig. 1) and clear cell RCC at age 35. The patient developed multiple skin lesions throughout her life. Additional diagnoses included primary hyperaldosteronism caused by bilateral adrenal hyperplasia, and reactive thrombocytosis (negative *JAK2*, *CALR*, and *MPL* somatic mutations) (ig. F).

### Family history

Family history revealed two deceased relatives with *BAP1*-TPDS-associated tumours:

1. *Individual II-2*: atypical meningioma at 44 years; aggressive clinical course with two postoperative recurrences, resulting in terminal disease. Additional medical history of splenectomy for splenomegaly at 36 years; splenic histology showed extramedullary haematopoiesis with myeloid-lineage expansion.
2. *Individual I-2*: RCC at age 62 (histological subtype unknown).

See Fig. 8 for a limited pedigree.

### BAP1 IHC

IHC with BAP-1 (BRCA1-Associated Protein 1) antibody, clone C-4, from Santa Cruz Biotechnology (Texas, USA) was performed on FFPE tissue slides from the patient’s RCC, splenic hamartoma, uterine leiomyoma, and three skin lesions, and the first-degree relative’s spleen. IHC with CD21 and CD34 antibodies was also performed on the first-degree relative’s spleen.

### Genetic testing

Genomic DNA was extracted from peripheral blood using the QIAamp DNA Mini Kit (QIAGEN). The extracted DNA was sent to Fulgent Genetics (California, USA) for Comprehensive Cancer Panel analysis. A buccal swab was also sent to deCODE Genetics (Reykjavik, Iceland) for whole-genome sequencing. Variant classification based on the ACMG/AMP guidelines for classification of sequence variants was performed locally [8]. The online variant evaluation tool Franklin by Genoox was used [9]. AlphaMissense (Google DeepMind) was among the in silico tools applied to predict missense‐variant pathogenicity [10].

## Results

### BAP1, CD21, and CD34 IHC results

**Fig. 2:**
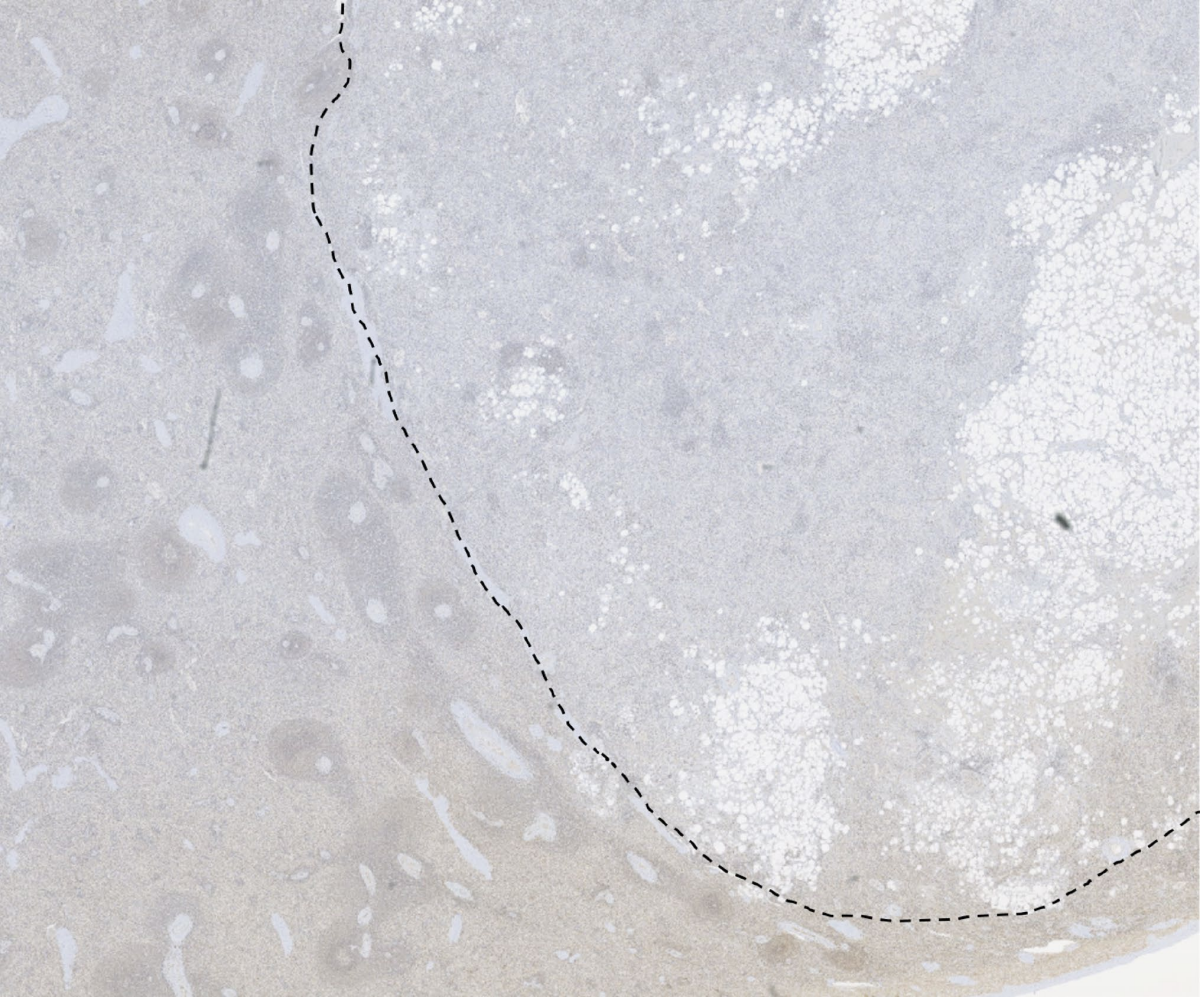
BAP1 IHC of spleen (low-power), patient. The dotted line marks the approximate boundary between normal splenic tissue (left) and the hamartoma (right)

**Fig. 3:**
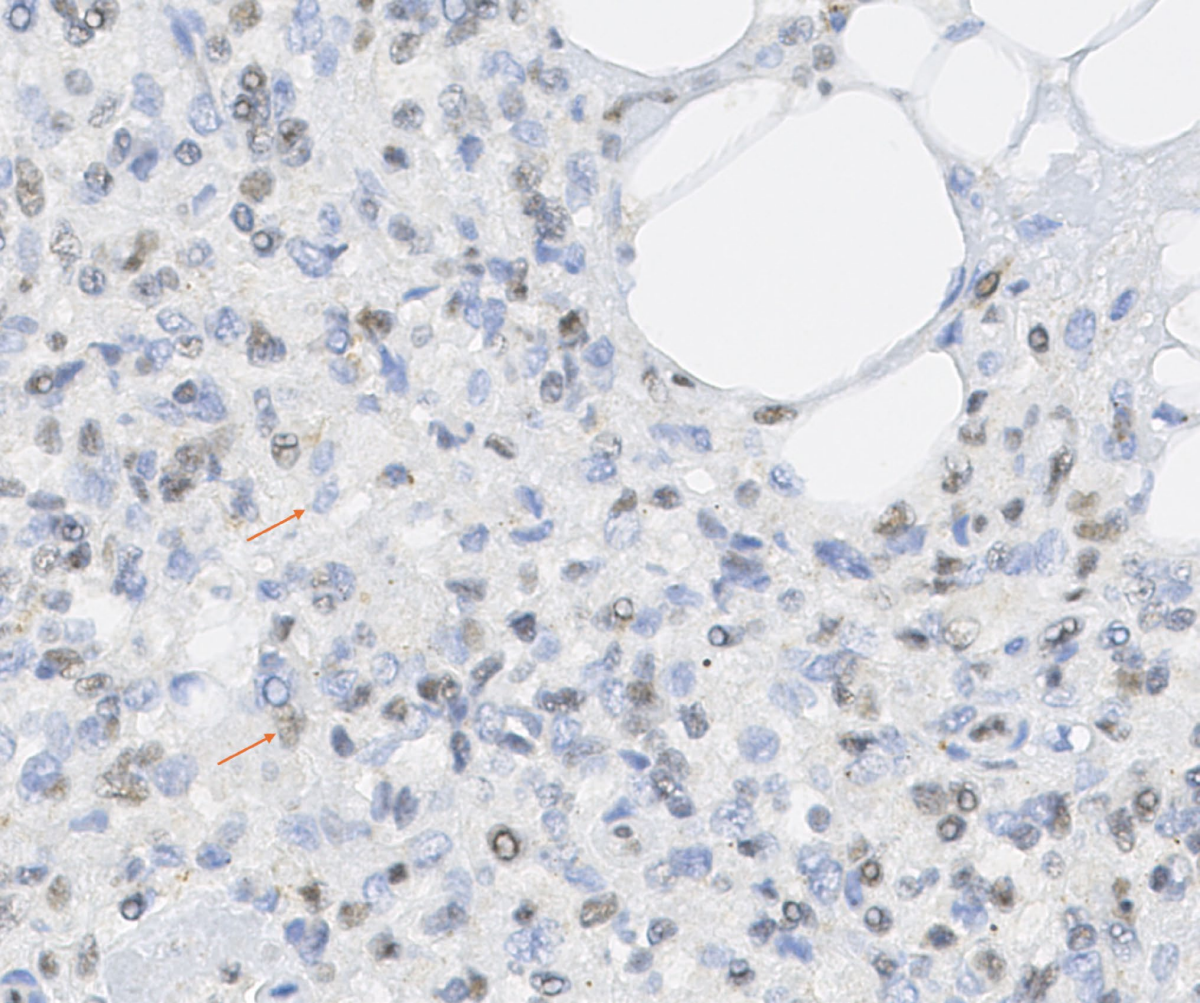
BAP1 IHC of splenic hamartoma (high-power), patient. Upper arrow: hamartoma cell with absent BAP1 nuclear staining. Lower arrow: non-hamartomatous cell with preserved BAP1 nuclear staining

**Fig. 4:**
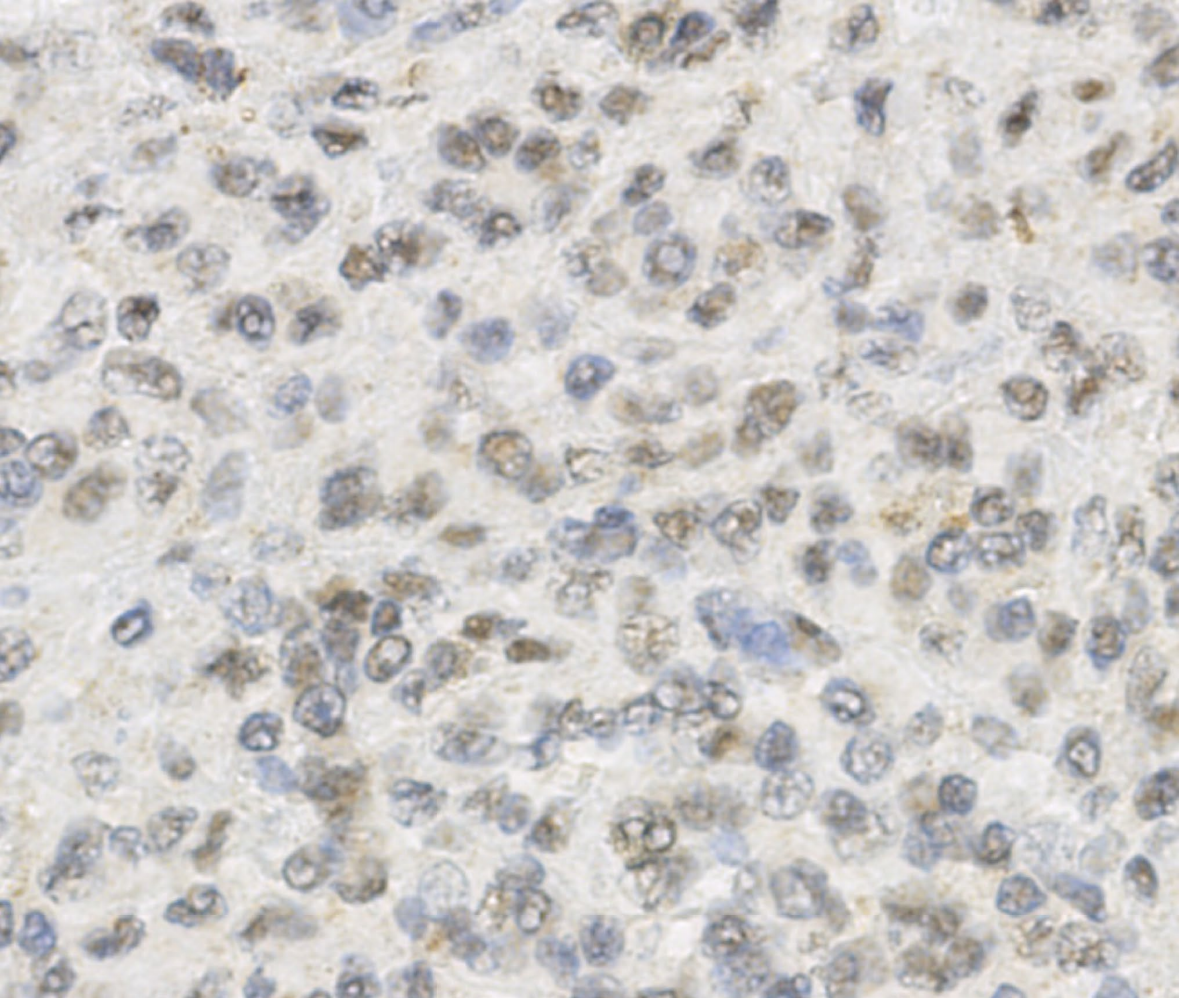
BAP1 IHC of spleen (high-power), patient

**Fig. 5:**
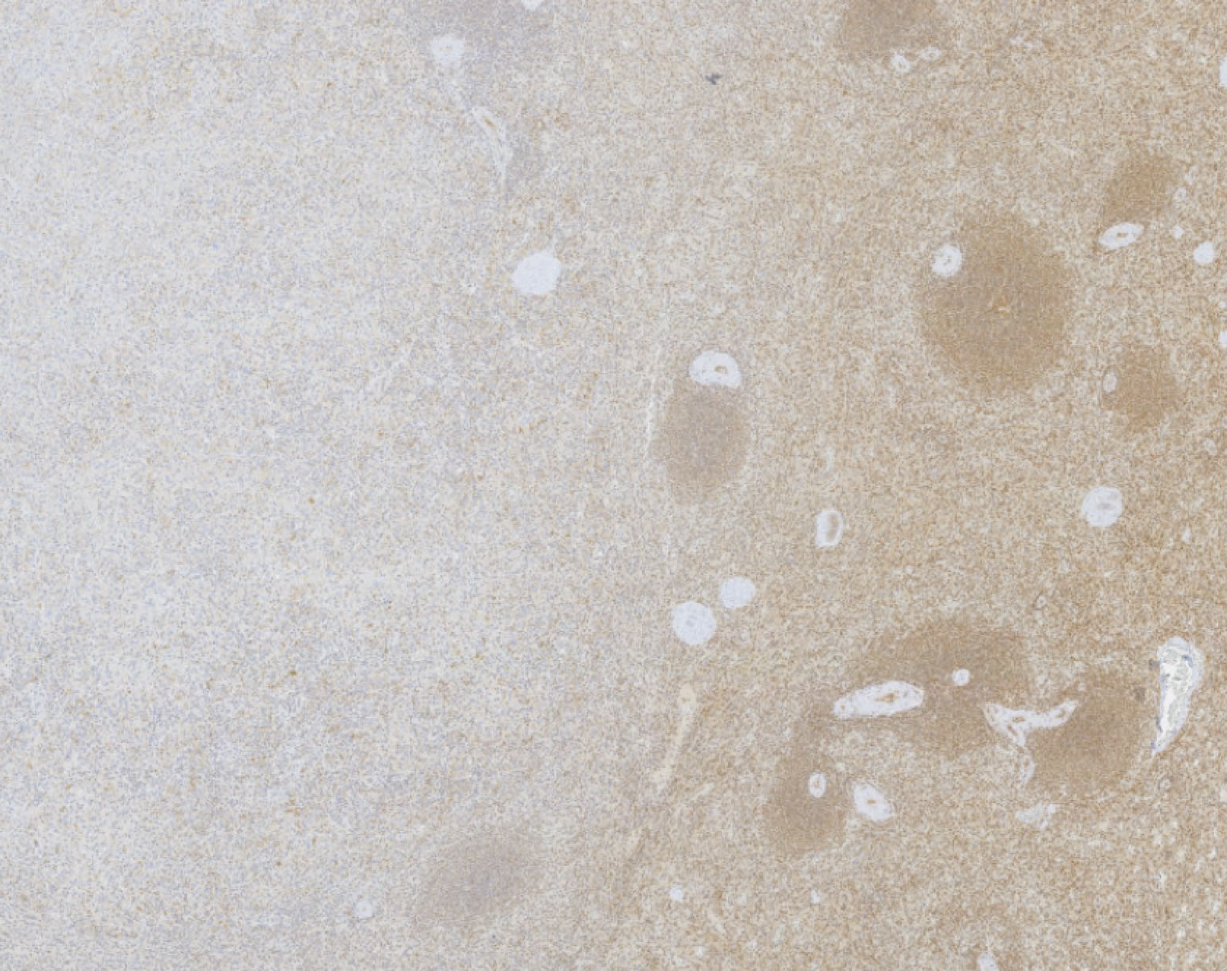
BAP1 IHC of spleen (low-power), individual II-2. Right: non-hamartomatous splenic tissue with retained BAP1 nuclear staining. Left: hamartoma with clearly less BAP1 staining. Note the presence of lymphoid follicles in the non-hamartomatous splenic tissue but not in the hamartoma

**Fig. 6:**
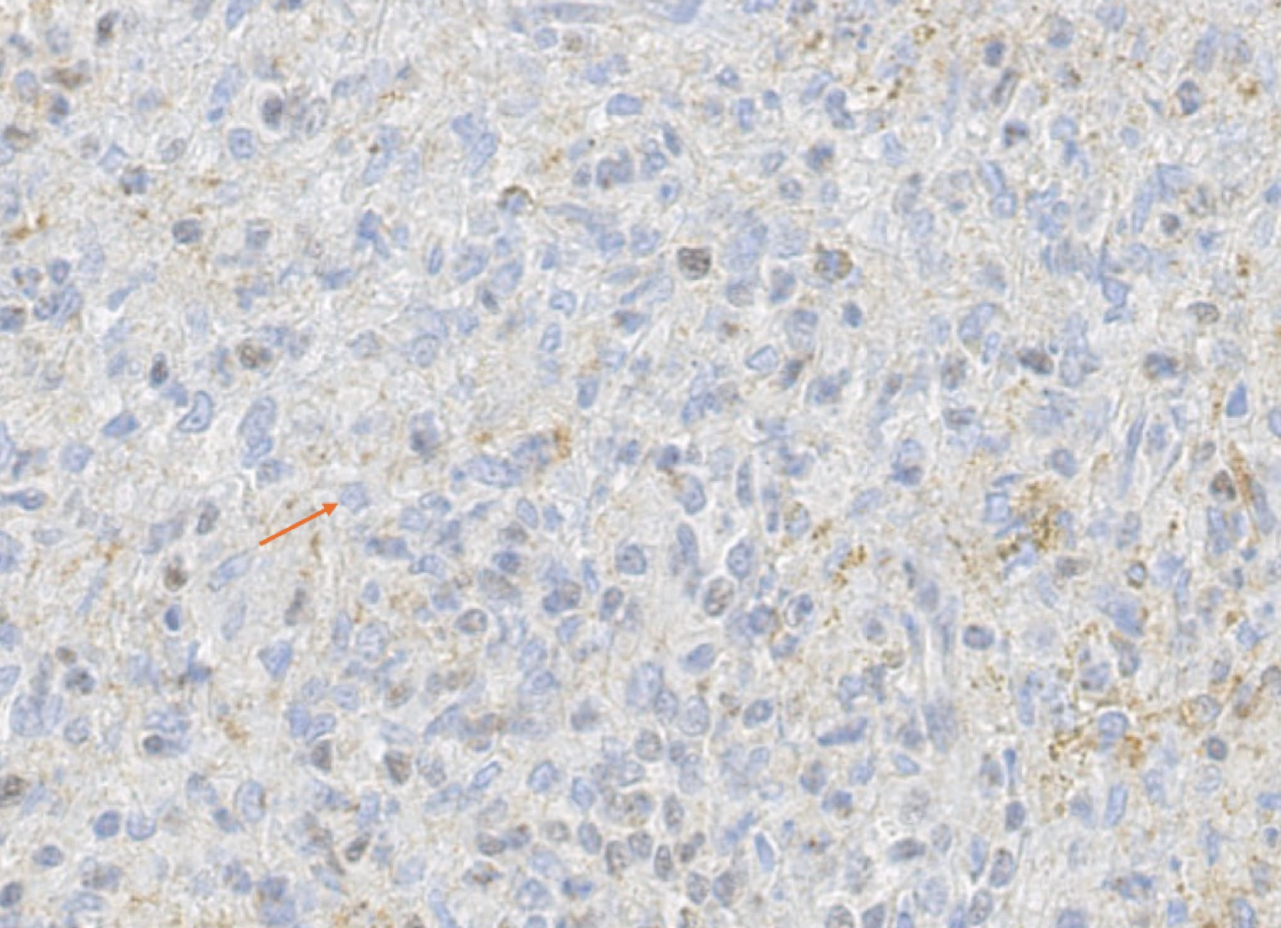
BAP1 IHC of spleen (high-power), individual II-2. Arrow: hamartoma cell with absent BAP1 nuclear staining

**Fig. 7:**
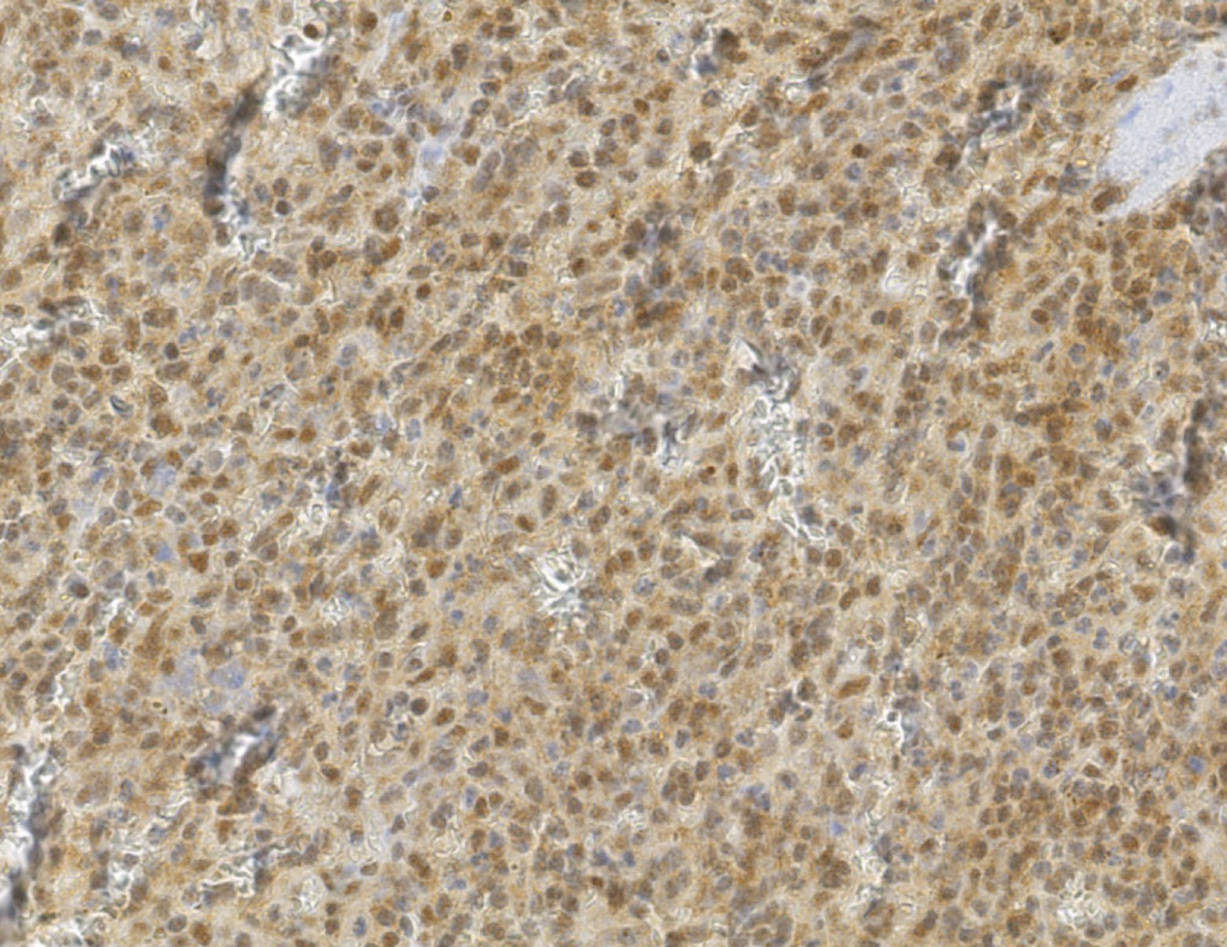
BAP1 IHC of non-hamartomatous splenic tissue (high-power), individual II-2. Retained BAP1 nuclear staining

The IHC results are shown in Table 1 and Figs. 2, 3, 4, 5, 6 and 7 (Figs. 2 and 3, patient; Figs. 4, 5, 6 and 7, individual II-2). Brown nuclear coloration indicates preserved BAP1 nuclear staining; absence of nuclear staining indicates BAP1 loss.

**Table 1:** IHC results

Sample	BAP1 nuclear staining	Note
Skin lesions (patient)	Absent	Histopathological features were consistent with BIMTs. Diagnosed as BIMTs
Renal cell carcinoma (patient)	Absent	*BAP1*-TPDS core tumour
Splenic hamartoma (patient)	Absent in a subset of cells	Figures 2, 3 and 4. Non-hamartomatous splenic tissue showed retained BAP1 nuclear staining. See Discussion
Uterine leiomyoma (patient)	Present	Not a *BAP1*-TPDS-associated tumour. Presumably sporadic
Splenic hamartoma (individual II-2)	Absent in a subset of cells	Re-evaluation of the splenic sample revealed an incidental splenic hamartoma with absent BAP1 nuclear staining in a subset of cells (Fig. 4). Non-hamartomatous splenic tissue showed retained BAP1 nuclear staining (Fig. 5). CD34 and CD21 staining patterns were consistent with splenic hamartoma (supplementary material). See Discussion

IHC, immunohistochemistry; BIMTs, *BAP1*-inactivated melanocytic tumours; *BAP1*-TPDS, *BAP1* tumour predisposition syndrome

### Genetic testing results

**Fig. 8:**
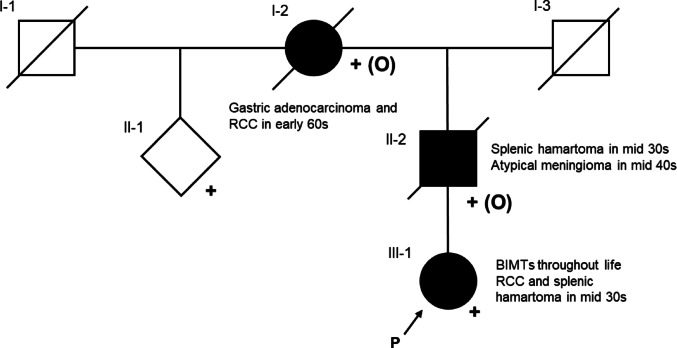
Pedigree depicting segregation of *BAP1* c.382G > A, p.(Gly128Arg). Proband, III-1. Heterozygous individuals whose samples were available for testing are indicated with + ; obligate carriers are indicated with + (O). Only the relatives whose genotypes were informative with regards to the obligate carrier status of I-2 and II-2 are included. II-1 not examined due to lack of accessibility

Genetic testing identified a heterozygous *BAP1* variant, NM_004656.4:c.382G > A, p.(Gly128Arg). The variant is absent from gnomAD v4.1.0. Among approximately 70,000 whole-genome sequenced Icelanders at deCODE genetics, there are only two carriers of the variant, the patient and a close relative. Their common ancestor was born in the 1920s, indicating that the variant is private to the family. Segregation analysis showed that the affected relatives were obligate carriers (see Fig. 8). The authors classified the variant as likely pathogenic (ACMG/AMP criteria PS3_supporting, PM1, PM2_supporting, PP1, PP3, and PP4; see Supplementary Material). The variant was submitted to ClinVar (submission ID: SUB15454254). *BAP1*-TPDS surveillance was initiated and cascade testing was offered to at-risk relatives. In addition to the *BAP1* variant, a heterozygous missense variant in the *BLM* gene, NM_000057.3:c.191A > T, p.(Asp64Val), was reported in the cancer panel where it was classified as a variant of uncertain significance. Biallelic pathogenic variants in the *BLM* gene are associated with autosomal recessive Bloom syndrome (OMIM #210900). The *BLM* variant was not considered to explain the patient’s phenotype. No other variants were reported in the WGS.

## Discussion

The p.Gly128Arg variant in *BAP1* has been reported as a somatic variant in uveal melanoma and peritoneal mesothelioma [11–13]. Our finding demonstrates that in addition to this, it is a disease-causing germline variant that leads to *BAP1*-TPDS. Our segregation of the variant within the patient’s family has confirmed its germline status. The occurrence of splenic hamartomas in two related individuals with *BAP1*-TPDS is consistent with emerging evidence that benign splenic lesions can be associated with *BAP1*-TPDS [5].

### The ubiquitin carboxy-terminal hydrolase (UCH) domain of the BAP1 protein

The p.Gly128Arg variant is located in the UCH domain (amino acids 1–240) of the BAP1 protein. The UCH domain is responsible for BAP1’s deubiquitinating activity. Glycine, a hydrophobic residue, is replaced by arginine, a large polar charged residue. This would be expected to disrupt the integrity of the UCH domain presumably leading to loss of its function [14]. Waters et al. (2024) used saturation genome editing in a *BAP1*-dependent haploid cell model to functionally characterize 18,108 unique *BAP1* variants. Deleterious variants reduced cell fitness and were classified as functionally depleted. The p.Gly128Arg variant was classified as functionally depleted [15]. Walpole et al. (2018) identified 36 unique germline *BAP1* missense variants in a review of *BAP1* variant-carrying families. The authors classified nine of the 36 missense variants as likely pathogenic. All nine variants were in the UCH domain of BAP1, consistent with it being a critical domain [3].

### Splenic hamartoma in *BAP1*-TPDS

Splenic hamartomas are rare. The reported incidence rate of splenic hamartomas in autopsy series is 0.024–0.13%. Loss of BAP1 expression on IHC is not a typical feature of splenic hamartomas.

Splenic hamartomas are not well demarcated microscopically and typically contain an admixture of hamartoma cells and non-hamartomatous cells [16].

The occurrence of splenic hamartomas in two related individuals is highly suggestive of an underlying shared genetic predisposition. Furthermore, our finding of loss of BAP1 expression on IHC in a subset of cells in both splenic hamartomas suggests that *BAP1*-TPDS represents this shared genetic predisposition. This is consistent with emerging evidence indicating that benign splenic lesions, including a suspected splenic hamartoma based on imaging, occur in *BAP1*-TPDS [5]. Further research is needed to determine how common splenic hamartomas are in *BAP1*-TPDS.

### *BAP1* and haematopoiesis

Postnatal *Bap1* knockout in mice causes myelodysplasia, myeloid skewing, ineffective haematopoiesis, and splenomegaly caused by extramedullary haematopoiesis and myeloid lineage expansion [17]. Dual *Bap1*/*Ezh2* knockout does not cause the phenotype, indicating the myeloid expansion is Ezh2-dependent [18].

This data suggests that there is a link between the obligate-carrier relative’s splenomegaly (caused by extramedullary haematopoiesis and myeloid lineage expansion) and *BAP1*-TPDS. While hypothesis-generating, this preliminary observation of a single case requires further corroboration in additional patients with *BAP1*-TPDS.

### *BAP1*-altered meningioma

Meningioma is among the less common *BAP1*-TPDS-associated tumours [3]. Somatic *BAP1* variants are known to occur in meningioma. *BAP1*-altered meningiomas represent < 1% of all meningiomas, but a small case series showed that 50% arose in the setting of *BAP1*-TPDS [19]. *BAP1*-altered meningioma has been suggested to represent a distinct and aggressive CNS tumour subtype [20]. Although BAP1 IHC was not performed on the meningioma of the patient’s obligate-carrier relative, its aggressive clinical course is consistent with what has been reported on *BAP1*-altered meningioma.

## Conclusion

We report two confirmed splenic hamartomas with loss of BAP1 in a subset of cells on IHC, in two related individuals with *BAP1*-TPDS. This case report adds NM_004656.4(*BAP1*):c.382G > A, p.(Gly128Arg) to the list of reported germline *BAP1* missense variants that cause *BAP1*-TPDS and supports emerging evidence indicating an association between splenic hamartoma and other benign splenic lesions to *BAP1*-TPDS. Furthermore, it demonstrates the utility of BAP1 IHC in the evaluation of germline *BAP1* variants. The patient’s bilateral adrenal hyperplasia and reactive thrombocytosis were presumably coincidental, as there are currently no known associations between these clinical features and *BAP1*-TPDS.

## Supplementary Information

Below is the link to the electronic supplementary material.

Supplementary Material 1

## Data Availability

The data that support the findings of this study are available from the corresponding author upon request. The detected BAP1 variant was submitted to ClinVar (reserved accession SCV006277940).
