# Supplementary material for: Splenic hamartoma in two related patients with BAP1 tumour predisposition syndrome caused by a novel germline BAP1 p.(Gly128Arg) missense variant
Source: Fam Cancer. 2026 Feb 19;25(1):23. doi: 10.1007/s10689-026-00538-3 (PMC12920776; doi:10.1007/s10689-026-00538-3)
Supplement: Supplementary file 1 — Supplementary Material 1 [file 10689_2026_538_MOESM1_ESM.docx]

**Supplementary Material** — Splenic hamartoma in two related patients with *BAP1* tumour predisposition syndrome caused by a novel germline *BAP1* p.(Gly128Arg) missense variant

Variant classification

Variant classification was performed according to the ACMG/AMP guidelines [(1)](https://sciwheel.com/work/citation?ids=632413&pre=&suf=&sa=0).

**Gene symbol**  *BAP1*

**Transcript**  NM_004656.4:c.382G>A

**Protein**  NP_004647.1:p.(Gly128Arg)

**Chromosomal GRCh38** NC_000003.12:g.52407454C>T

**Classification:** Likely pathogenic

- **PS3_supporting (functional studies)** — Saturation genome editing of *BAP1* variants classified the p.Gly128Arg variant as functionally depleted [(2)](https://sciwheel.com/work/citation?ids=16639762&pre=&suf=&sa=0), see Discussion.
- **PM1 (functional data)** — The variant is in the ubiquitin carboxy-terminal hydrolase domain (amino acids 1–240) of BAP1. The UCH domain is a critical domain and is responsible for BAP1’s deubiquitinating activity [(3)](https://sciwheel.com/work/citation?ids=9270361&pre=&suf=&sa=0).
- **PM2_supporting (population data)** — Absent from the Genome Aggregation Database (gnomAD) [accessed June 21, 2025] [(4)](https://sciwheel.com/work/citation?ids=8969305&pre=&suf=&sa=0).
- **PP1 (segregation data)** — Familial segregation studies revealed that the two deceased relatives mentioned in 2.1 were obligate carriers. See Discussion on meningioma and *BAP1*.
- **PP3 (*in silico* predictions)** — Unanimous prediction of deleterious effect; AlphaMissense gave the variant the maximum pathogenicity score, 1.0 [(5,6)](https://sciwheel.com/work/citation?ids=15380065,17987344&pre=&pre=&suf=&suf=&sa=0,0).
- **PP4 (phenotype)** — The patient’s tumor phenotype is highly specific for *BAP1*-TPDS: multiple BIMTs and a clear cell RCC, all with loss of nuclear BAP1 staining on IHC.


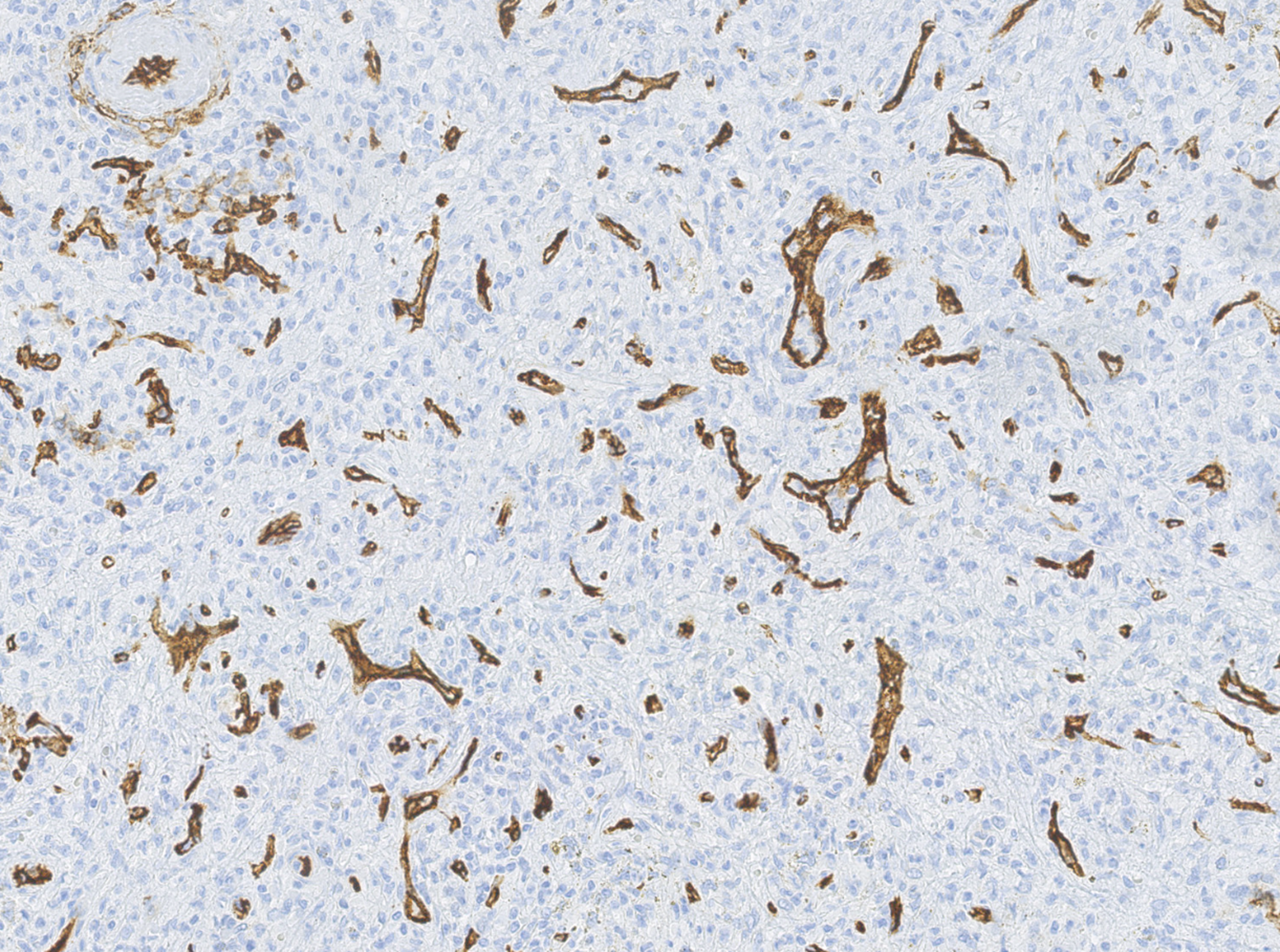


**Supplementary fig. 1** CD34 IHC of splenic hamartoma (high-power), individual II-2. Capillaries show CD34 staining; hamartoma cells are CD34-negative


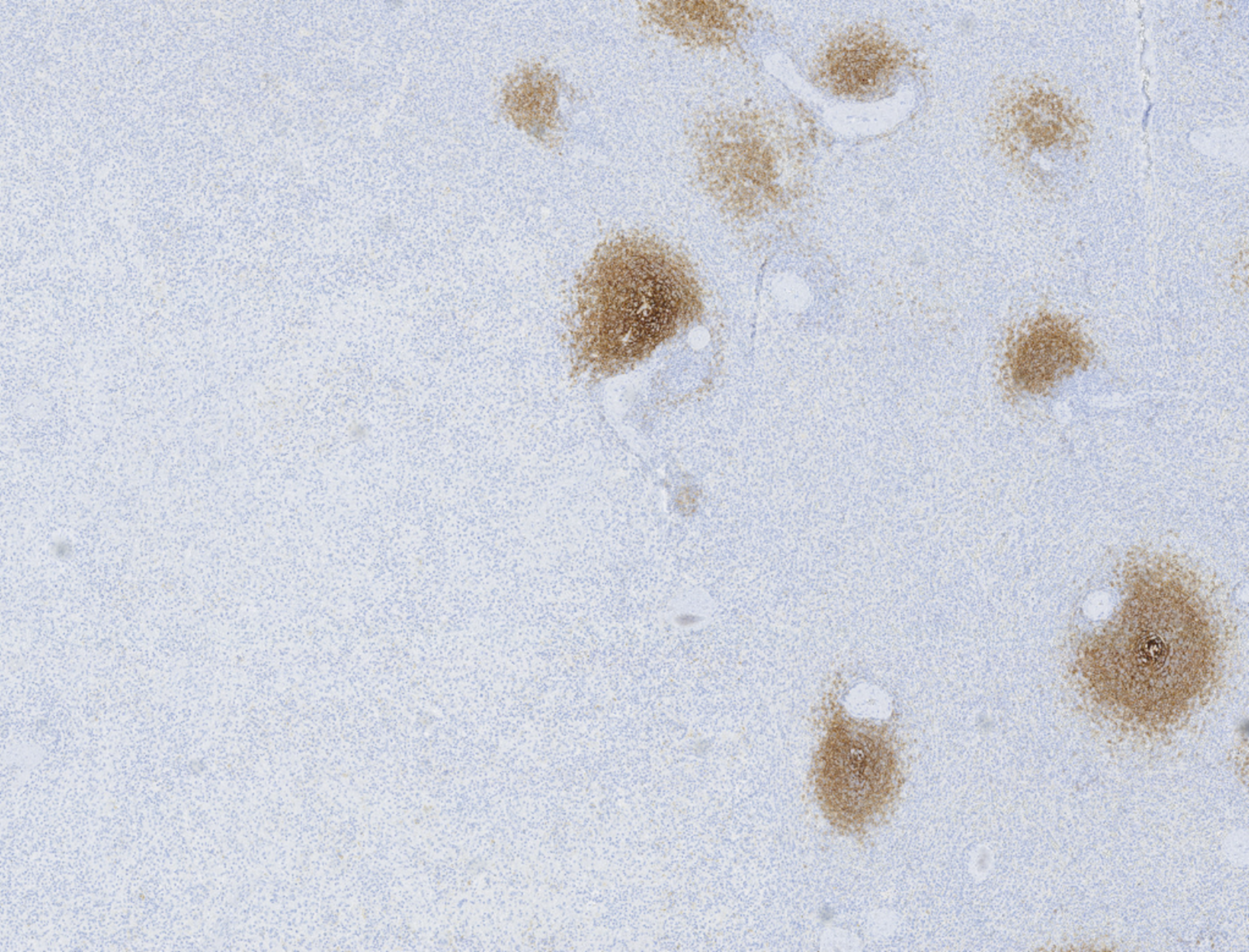


**Supplementary fig. 2** CD21 IHC of spleen (high-power), individual II-2. Right: non-hamartomatous splenic tissue with CD21 staining in lymphoid follicles. Left: hamartoma showing absence of CD21 staining

**REFERENCES**

[1. Richards S, Aziz N, Bale S, Bick D, Das S, Gastier-Foster J, et al. Standards and guidelines for the interpretation of sequence variants: a joint consensus recommendation of the American College of Medical Genetics and Genomics and the Association for Molecular Pathology. Genet Med. 2015 May;17(5):405–24.](https://sciwheel.com/work/bibliography/632413)

[2. Waters AJ, Brendler-Spaeth T, Smith D, Offord V, Tan HK, Zhao Y, et al. Saturation genome editing of BAP1 functionally classifies somatic and germline variants. Nat Genet. 2024 Jul 5;56(7):1434–45.](https://sciwheel.com/work/bibliography/16639762)

[3. Sharma A, Biswas A, Liu H, Sen S, Paruchuri A, Katsonis P, et al. Mutational Landscape of the BAP1 Locus Reveals an Intrinsic Control to Regulate the miRNA Network and the Binding of Protein Complexes in Uveal Melanoma. Cancers (Basel). 2019 Oct 19;11(10).](https://sciwheel.com/work/bibliography/9270361)

[4. Karczewski KJ, Francioli LC, Tiao G, Cummings BB, Alföldi J, Wang Q, et al. The mutational constraint spectrum quantified from variation in 141,456 humans. Nature. 2020 May 27;581(7809):434–43.](https://sciwheel.com/work/bibliography/8969305)

[5. Cheng J, Novati G, Pan J, Bycroft C, Žemgulytė A, Applebaum T, et al. Accurate proteome-wide missense variant effect prediction with AlphaMissense. Science. 2023 Sep 22;381(6664):eadg7492.](https://sciwheel.com/work/bibliography/15380065)

[6. Franklin by Genoox [Internet]. [cited 2025 Jun 19]. Available from: https://franklin.genoox.com](https://sciwheel.com/work/bibliography/17987344)
